# Effects of Fermentation Time and Temperature on the Physicochemical Quality of Kombucha

**DOI:** 10.3390/foods15071226

**Published:** 2026-04-03

**Authors:** Juan Pablo Salinas-Ruiz, Jesús Guevara García, Daniela Rios Tovar, Reichell P. Cruz Cabrera, Damir D. Torrico

**Affiliations:** 1 Department of Food Science and Human Nutrition, University of Illinois Urbana-Champaign, Urbana, IL 61801, USA; a01177749@exatec.tec.mx (J.P.S.-R.); jessag2@illinois.edu (J.G.G.); dr41@illinois.edu (D.R.T.); reichell@illinois.edu (R.P.C.C.); 2 Escuela de Ingeniería y Ciencias, Tecnológico de Monterrey, Ave. Eugenio Garza Sada 2501, Monterrey 64849, Nuevo León, Mexico

**Keywords:** kombucha, fermentation conditions, pH, viscosity, response surface methodology

## Abstract

Kombucha is a fermented tea beverage produced through the metabolic activity of a symbiotic culture of bacteria and yeasts (SCOBY). Although consumer demand for kombucha has increased substantially, the influence of fermentation conditions on product quality remains insufficiently understood. This study evaluated the effects of fermentation temperature and time on the physicochemical properties of kombucha. A total of 20 L of kombucha was prepared using black tea (10 g/L) and sucrose (70 g/L). After filtration, the mixture was adjusted to pH 4.14 and inoculated with SCOBY. Fermentations were conducted at three temperatures (23, 25, and 28 °C) and at three time points (7, 11, 15 days). Following fermentation, pH, viscosity, soluble solids (°Brix), titratable acidity, color, and concentrations of lactic and acetic acids were quantified. The main results showed that pH decreased progressively with increasing fermentation temperature and time (from 3.47 at 23 °C/7 days to 2.96 at 28 °C/15 days). Concentrations of lactic and acetic acids increased with fermentation time, consistent with fermentation progression. Response surface modeling (RSM) indicated nonlinear interactions between time and temperature for pH and viscosity. Overall, the results identified fermentation parameters that enhanced desirable kombucha attributes, providing a scientific basis for formulation and process optimization in commercial production.

## 1. Introduction

Kombucha, or fungus tea, is a fermented beverage prepared using a symbiotic culture of bacteria and yeasts (SCOBY) [1,2]. In recent years, the demand for kombucha has grown rapidly. The global market size in 2018 was $1.5 billion and is projected to reach $5 billion by 2025 [3]. The SCOBY in kombucha is primarily composed of acetic acid bacteria (AAB), yeast, and, in some cases, lactic acid bacteria (LAB). These microorganisms are frequently described as a microbial community that may contribute to functional attributes for gut health; however, this depends on strain, identity, viability, and dose [3,4]. In the fermentation process, the SCOBY forms a solid cellulosic pellicle at the surface of the liquid. This bacterial cellulose (BC) is produced by AAB, normally from the *Gluconacetobacter* genus (Gram-negative bacteria), and supports the growth of all microorganisms in SCOBY during fermentation [5,6]. BC develops from a glucose-based polymer that promotes uridine diphosphate glucose monomers to form cellulose fibers [7].

During fermentation, yeast in SCOBY hydrolyzes sucrose into glucose and fructose through invertase activity. These monosaccharides are subsequently fermented into ethanol and carbon dioxide. Then, AAB oxidize ethanol to acetic acid and convert glucose into organic acids, including gluconic and glucuronic, which contribute to the characteristic acidity and chemical complexity of kombucha [3,8]. While these organic acids have been suggested to play roles in metabolism and detoxification pathways, their specific physiological effects in humans remain insufficiently characterized [9].

On the other hand, black tea, the primary substrate for kombucha production, contains a diverse array of polyphenolic compounds, including theaflavins, thearubigins, gallic acid, and other flavonoids, which have demonstrated antioxidant and various bioactive properties in experimental and preclinical studies [10]. During the manufacture of black tea, catechins undergo enzymatic oxidation to form theaflavins, thearubigins, and higher-molecular-weight theabrownins. During kombucha fermentation, further biotransformation of tea polyphenols can occur, although the direction and magnitude of changes in antioxidant activity vary among studies and depend on factors such as tea grade, processing conditions, and fermentation parameters [10].

Despite their potential nutraceutical properties and their unique sensory characteristics, very few studies have evaluated the effects of different processing techniques on the physicochemical and quality characteristics of kombucha products. As fermentation processes can profoundly affect these characteristics, relatively little is still known about how fermentation parameters modify the properties of kombucha. For instance, a previous study revealed that the physicochemical characteristics of kombucha, such as acidity and turbidity, increased with extended fermentation times [1]. However, there is still a lack of understanding about the interaction between time and temperature during fermentation and its impact on the key quality parameters of kombucha.

Variables such as the concentration and composition of microorganisms in SCOBY, the sugar content, tea type, and fermentation conditions, including temperature and duration, influence the nutritional value and taste of kombucha [11]. Literature suggests that a sucrose concentration of 70 g/L is optimal for higher sugar uptake and increased lactic acid production in kombucha fermentation [12]. The optimal temperature for kombucha fermentation ranges from 20 to 30 °C, with 25 °C producing the highest glucuronic acid concentration. Temperature is considered another key factor influencing the quality parameters in kombucha [2]. Therefore, the temperature range (23–28 °C) for this study was chosen because it has been reported as optimal for kombucha fermentations. The selected times (7, 11, and 15 days) represent the early, intermediate, and extended stages of kombucha fermentation, since fermentation is usually conducted for 10–14 days [2,13,14,15]. This research aims to determine how fermentation time and temperature affect the physicochemical quality attributes of kombucha during fermentation, to better understand their combined effects, and to support the optimization of the fermentation process.

## 2. Materials and Methods

### 2.1. Materials

The materials employed in the kombucha preparations included water (Great Value^TM^, Walmart Inc., Bentonville, AR, USA), granulated sugar (Great Value^TM^), and Harney & Sons Chinese Black Tea (Millerton, NY, USA). Additionally, SCOBYs and starter culture from the brand Fermentaholics (Clearwater, FL, USA) were used to initiate the fermentation process. The microbial composition of the SCOBY used in this study was characterized by the supplier using 16S ribosomal DNA sequencing, revealing a diverse community of bacteria, including *Acetobacter tropicalis* (99% confidence of detection), *Bacillus* sp. aerophilus (96%), *Bacillus* sp. aryabhattai (98%), *Bacillus* sp. cereus (99%), *Bacillus* sp. licheniformis (99%), *Bacillus* sp. pumilus/aerophilus/safensis/altitudinis (99%), *Bacillus* sp. subtilis (99%), *Gluconacetobacter rhaeticus* (98%), *Gluconacetobacter saccharivorans* (99%), *Micrococcus* sp. (98%), and *Paenibacillus taichungensis* (97%). The yeast population was composed of *Brettanomyces bruxellensis* (99%), *Candida* spp. (97%), *Saccharomyces cerevisiae* (92%), and *Zygosaccharomyces* spp. (97%).

The fermentation process was conducted in mason jars of 900 mL, which were covered with cheesecloth to permit air flow and protection. The fabric allows oxygen to enter, which is essential for kombucha’s aerobic fermentation, while acting as a barrier to prevent contamination. Controlled fermentation conditions were maintained using an Isotemp Incubator (Fisher Scientific, Waltham, MA, USA) and a C76 Water Bath Shaker (New Brunswick Scientific, Edison, NJ, USA). For analytical measurements, several instruments were utilized, including an NDJ-5S Viscometer (Bonvoisin, Shanghai, China), a Soonda WR-10 Colorimeter (DahoMeter, Dongguan, China) for color assessment, a PH700 pH meter (APERA, Columbus, OH, USA), a PAL-30S acetic acid pocket refractometer (ATAGO^®^, Tokyo, Japan), and a PAL-Easy ACID96 lactic acid pocket refractometer (ATAGO^®^). Density, °Brix, and alcohol content were measured with an EasyDens (Anton Paar, Graz, Austria), used alongside its Brew Meister and Proof Meister apps for precision measurement. The initial and final weights for each SCOBY were determined with AP210 analytical plus scale (OHAUS, Parsippany, NJ, USA).

### 2.2. Kombucha Preparation

For the kombucha preparation, a total of 20 L of kombucha were prepared, containing a concentration of 10 g tea/L and sugar at 70 g/L [12,13]. The water was heated to 95 °C, and the temperature was monitored with a Digital Thermometer Model 4400 (Ertco-Eutechincs, Alpha Technics, Irvine, CA, USA) to maintain consistency [2]. Upon reaching 95 °C, the heat was turned off, and the tea was added, followed by 10 min stirring for steeping [3,13]. Subsequently, the tea leaves were filtered, and sugar was incorporated into the mixture. As some water evaporated during the heating process, additional water (~150 mL) was added to maintain the desired volume (20 L).

After cooling, the tea infusion (initial pH 5.15) was adjusted to pH 4.14 by incorporating liquid from the starter culture following the manufacturer’s instructions (Fermentaholics). This acidification step was performed to ensure microbiological safety of the kombucha beverage [8]. To promote oxygenation, the mixture was transferred repeatedly between sterile containers prior to dispensing 800 mL into each mason jar. Each SCOBY was weighed before inoculation to record its initial mass and then added to the tea infusions. Jars were then covered with sterile cheesecloth and secured with rubber bands to minimize external microbial contamination while allowing airflow. The temperature range selected in this study was chosen since it is commonly reported in kombucha fermentations as optimal. Fermentations were conducted in duplicate at three temperatures (23, 25, and 28 °C) and three incubation periods (7, 11, and 15 days), yielding nine experimental treatment combinations [2,8,13,14].

### 2.3. Analytical Measurements Methodology

Upon completing the fermentation process, different analytical measurements were conducted to evaluate the development of the kombucha. Each sample’s pH was recorded. The viscosity of the samples was determined using a viscometer set at 30 RPM with a #0 rotor, analyzing 30 mL of each sample for 1 min. To determine total acid content, a 5 mL sample was titrated using 0.025 M NaOH as the titrant. The titration was conducted until the pH reached 8.2 [13]. The volume of NaOH used was recorded and employed to calculate the total acid content of each sample. Measurements of density (g/cm^3^), Brix degrees, and alcohol content (% *v*/*v*) were performed using the EasyDens instrument. The equipment was cleaned with alcohol and distilled water before use to eliminate residues. Each sample (~2 mL) was passed through the system and recorded. Sample color was evaluated using a calibrated colorimeter against a white background. Finally, each SCOBY was weighed at the end of the fermentation to compare the weight before and after the process.

### 2.4. Statistical Analysis

The experiment was designed using a factorial treatment arrangement with a two-way Analysis of Variance (ANOVA) that included three fermentation temperatures (23, 25, and 28 °C) and three incubation periods (7, 11, and 15 days), resulting in a total of nine treatments. Each treatment was replicated twice, leading to 36 experimental units. The 3 × 3 factorial design (temperature × time) was selected to systematically evaluate the interaction between the two independent variables. Data were analyzed using an alpha = 0.05. A post hoc Tukey’s honest multiple range test was conducted to compare the different kombucha products using RStudio (Version 2023.12.1, Boston, MA, USA). Additionally, for pH, Brix (°), acidity (mL), and viscosity (cP), response surface methodology (RSM) was applied to fit a second-order polynomial equation, Y = b_0_ + b_1_X_1_ + b_2_X_2_ + b_12_X_1_X_2_ + b_11_X_1_X_1_ + b_22_X_2_X_2_, where Y was the predicted response (pH, Brix, acidity or viscosity); b_0_ was the value of the fitted response at the center point of the design; b_1_ and b_2_ were linear regression terms; b_12_ was the cross product regression term; and b_11_ and b_22_ were quadratic regression terms. 3D contour plots were generated by R-studio. Furthermore, a principal component analysis (PCA) was applied to explore the multivariate relationships between physicochemical variables and different kombucha formulations. PCA was performed using centered and scaled data to account for the variance structure among samples, resulting in a product-attribute bi-plot that was used to visualize the two principal components in the model using R-Studio.

## 3. Results and Discussion

### 3.1. pH

As shown in Table 1, pH levels decreased gradually across all treatments over time (having an initial pH of 4.14 on day 0). After fermentation, the highest pH was recorded at 23 °C on day 7, with a pH of 3.47, while the lowest pH was observed at 28 °C on day 15, with a value of 2.96. In the experiment conducted by Jayabalan et al. (2007) [16], the highest concentrations of organic acids in kombucha were observed at 28 °C on day 15 of the fermentation process. The primary organic acids accumulated in the fermented product were acetic, lactic, glucuronic, and citric acids. These conditions coincided with the parameters that affected the kombucha product with the lowest recorded pH in the present study. Also, this is consistent with faster fermentation progressions at higher temperatures and longer times, which are commonly associated with increased accumulation of organic acids in kombucha. However, because microbial populations were not quantified during fermentation, any explanation of the biological mechanisms should be interpreted as inferential and derived from the physicochemical patterns observed rather than direct microbial evidence. During fermentation, *Saccharomyces cerevisiae* secretes an invertase (β-d-fructosidase) that hydrolyzes the sucrose into glucose and fructose; these monosaccharides are then transported by hexose transporters and metabolized into ethanol and CO_2_ [17]. Under high glucose conditions, the MIG1 (multicopy inhibitor of GAL gene expression) repressor inhibits SUC2, a major invertase gene, blocking sucrose hydrolysis and depriving AAB and LAB of fermentable substrates. However, environmental pressure such as osmotic or oxidative stress and low pH reduces MIG1 activity, allowing the action of SUC2 expression, even in the presence of glucose [18]. As a result, *S. cerevisiae* continues to break down sucrose, supplying carbon sources to acid-producing bacteria and thereby driving a further decrease in pH. However, invertase activity is pH-dependent; at pH 3 or below, it falls to about 60% and continues to decline with further acidification, slowing acid production, and ultimately stabilizing the pH [19].

Nevertheless, increasing the fermentation temperature also accelerates acidification, as seen in the results of the present study (Table 1). Acetobacter and most AABs exhibit optimal activity around 30 °C, where they synthesize pyrroloquinoline quinone (PQQ). These PQQs, in particular, PQQ-dependent alcohol dehydrogenase (PQQ-ADH) and PQQ-dependent aldehyde dehydrogenase (PQQ-ALDH), oxidize the ethanol produced by *Saccharomyces cerevisiae* into acetic acid, thereby decreasing the pH [20]. According to the Henderson–Hasselbalch equation, it is possible to estimate the degree of dissociation of a weak acid by relating the pH of the solution to the acid’s pKa using the formula:

(pH = pKa+ log_10_ ([A^−^]/[HA]))

By rearranging the equation, the ratio between the base [A^−^] and the undissociated acid [HA] can be determined. Since each molecule of HA that dissociates produces one A^−^ and one H^+^, the concentration of A^−^ directly reflects the number of protons released, making it possible to determine how much acid is effectively contributing to the measured pH.

**Figure 1:**
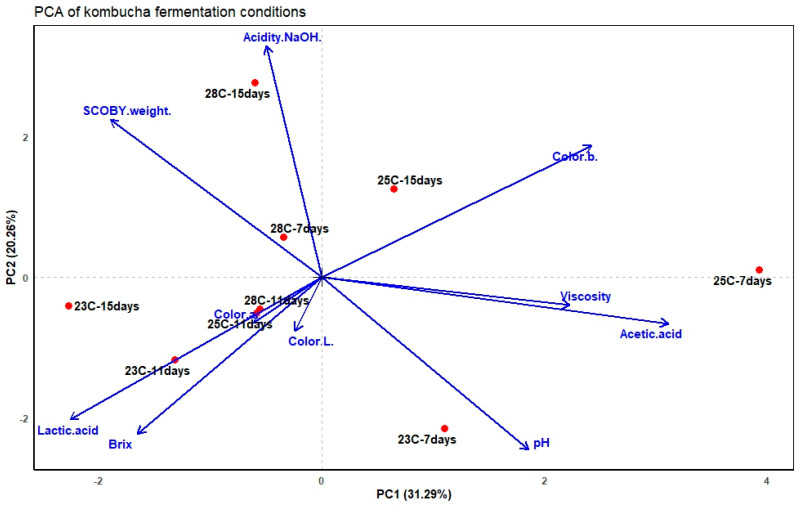
Principal component analysis (PCA) bi-plot visualizing treatments* (kombucha) and the physico-chemical/color parameters.

For example, based on the acetic acid pKa of 4.76, if the medium has a pH of 3, the ratio [A^−^]/[HA] equals approximately 0.174. This corresponds to [HA] = 98.29% and [A^−^] = 1.71%; around 1.71% of the acetic acid has dissociated and released H^+^, while 98.29% remains in the undissociated HA form. This allows us to quantify the portion of the acid that is actively influencing the pH and clarifies why the titratable acidity in Table 1 continues to increase substantially, even as the pH decline becomes less pronounced. When considering all the fermentation variables together, as shown in the PCA (Figure 1), the pH vector was aligned with the kombucha treatment at 23 °C for 7 days, which is in the opposite direction to the total acidity vector. This inverse relationship is expected since the production of acid lowers the pH. Additionally, higher pH values were observed during the early fermentation periods, indicating minimal organic acid accumulation at these time points.

### 3.2. Viscosity

At 23 and 25 °C, viscosity increased on day 7, decreased on day 11, and increased again on day 15. At 28 °C, viscosity was initially low, peaked on day 11, and then decreased by day 15. According to Cruz et al. (2024) [21], the viscosity of kombucha increases due to the accumulation of fibrous cellulose in the liquid infusion. Mainly, BC is biosynthesized through enzyme systems or aerobic culture using bacteria such as *Agrobacterium*, *Acetobacter*, *Gluconacetobacter*, *Komagataeibacter*, *Sarcina*, and *Pseudomonas* [22].

Normally, levels of dissolved oxygen are adequate at the beginning of the fermentation period; however, when cellulose forms a biofilm on the surface due to microbial growth, this affects the levels of dissolved oxygen [23]. These observations suggest that the rapid cellulose biosynthesis during the early stages of fermentation promotes the corresponding increase in viscosity, as shown in Table 1 for the 23 and 25 °C treatments. In contrast, the delayed viscosity increases observed in the 28 °C treatments indicate differences in pellicle development under elevated temperature conditions. Because cellulose and microbial content were not quantified, interpretation is limited to the observed physicochemical trends. As shown in Figure 1, viscosity was found to be associated with acetic acid, suggesting a potential biological connection.

### 3.3. Acetic Acid (%)

As shown in Table 1, the highest acetic acid concentration was observed at 25 °C on day 7, at 12.23%. In all treatments, levels declined slightly on days 11 and 15. At 28 °C, the acetic acid concentration remained somewhat stable. The most important factors influencing the growth of microorganisms are energy sources, temperature, and oxygen [24]. Low levels of dissolved oxygen in the matrix could affect microbial growth and metabolic activity. Since all AAB are aerobic and require oxygen to grow and survive, having low oxygen levels can affect their ability to oxidize ethanol into acetic acid.

Changes in acetic acid concentration over time and temperature potentially reflect the balance between ethanol oxidation and acid accumulation during aerobic fermentation. Oxygen limitation can emerge when carbon dioxide and ethanol accumulate beneath the pellicle, restricting microbial access to oxygen and nutrients, thereby reducing AAB and yeast and affecting acetic acid production [25]. As shown in Table 1, acetic acid (%) decreased on the 23 and 25 °C treatments, while lactic acid increased. This trend occurred with all kombucha products, except for the 28 °C treatments, where acetic acid (%) increased, and lactic acid (%) decreased. As shown in Figure 1, acetic acid was associated with the treatment at 25 °C for 7 days, suggesting a rapid AAB activity. In contrast, the lactic acid concentration increased as fermentation progressed during the 11- and 15-day periods.

### 3.4. Lactic Acid (%)

As observed in Table 1, lactic acid levels increased over time in the 23 and 25 °C treatments, peaking at 23 °C on day 15 with a value of 5.79%. The lowest concentration was observed at 25 °C on day 7 (1.79%). At 28 °C, lactic acid levels showed a fluctuation, initially increasing to a peak of 4.41% on day 11 and then decreasing to 2.30% by day 15. The lower concentrations of lactic acid compared to acetic acid may be attributed to the limited presence of lactic acid bacteria (LAB) within the microbial community of the kombucha starter culture. According to the microbial composition reported by Fermentaholics, none of the identified strains were classified as LAB. However, some Bacillus species present in the culture may contribute to lactic acid production under specific conditions. For instance, *Bacillus subtilis* has been reported to produce lactic acid under certain environmental conditions, as demonstrated by Garin-Murguialday et al. (2024) [26]. This suggests that the presence of lactic acid in the fermentation could be associated with metabolites produced by non-LAB. Further microbial tracking is needed to confirm these findings. As shown in Figure 1, the lactic acid concentration vector is in the opposite direction of the acetic acid concentration and SCOBY weight vectors, aligning with the treatments at 23 °C for 11 and 15 days. This indicates that low temperatures and longer fermentation times favor lactic acid production.

### 3.5. Brix (°)

As shown in Table 1, the Brix values decreased across treatments as fermentation progressed, reflecting the consumption of sugars by microorganisms. However, increases in Brix values were recorded at 23 °C on day 15, with 6.20°, and at 28 °C on day 15, with 5.75°. Refractometers do not specifically measure sucrose or glucose concentrations in solutions, but rather the overall refractive index of the medium based on dissolved solutes. As shown in Shehadeh et al. (2020) [27], glucose and fructose had the greatest effect on refractive index. However, it was also demonstrated that tartaric acid, glycerol, and ethanol affected the refractive index. Therefore, even as sugars are depleted, the accumulation of non-sugar compounds, such as ethanol, glycerol, organic acids (acetic, lactic, and glucuronic), and lysis-derived products like peptides and amino acids, can significantly influence °Brix readings during fermentation. This is supported by the titratable acidity (mL) values shown in Table 1, where many acids contribute to the soluble solids content measured by the refractometer. As shown in Figure 1, lower temperatures and shorter fermentation times appear to limit sugar consumption, explaining the higher °Brix values, which are associated with increased lactic acid concentrations. Ethanol and glycerol were not directly quantified in this study; therefore, their contribution to °Brix variability cannot be confirmed and is only presented as a known limitation of refractometric soluble solids in complex fermentations.

### 3.6. SCOBY Weight Change (g)

SCOBY mass increased progressively, with the largest gain at 28 °C on day 15, reaching 23.68 g. The increase in SCOBY weight observed in Table 1 at 28 °C potentially reflects a combination of pellicle/cellulose formation, water retention, and microbial biomass accumulation. Because bacterial cellulose was not quantified, SCOBY weight cannot be interpreted as a direct measure of BC yield, but it may serve as one of several contributing factors. Based on Njieukam et al. (2023) experiment [28], *Komagataeibacter*’s optimal temperature for BC production was 30 °C under static aerobic conditions. This bacterium belongs to the same group as *Acetobacter* and *Gluconacetobacter*, which are also well-known producers of BC in kombucha fermentations. This indicates that SCOBY biomaterial development was more efficient at 28 °C in the present study, reflecting the dependence of temperature in the process. As shown in Figure 1, the vector for SCOBY weight had a similar direction as titratable acid, indicating a possible association. This is consistent with the role of AAB in kombucha, which produces acetic acid and cellulose for SCOBY development; however, the specific contribution of water retention to biomass production cannot be separated without compositional measurements [22].

### 3.7. Acidity Titratable (mL)

Across all temperatures, titratable acidity, as shown in Table 1, has a high value on day 7, a noticeable decrease on day 11, and a pronounced increase on day 15. The most visible effect occurs at 28 °C, where acidity had a value of 23.10 mL after 7 days, decreased to 7.80 mL after 11 days, and increased to 25.15 mL after 15 days of fermentation. Villarreal-Soto et al. (2018) reported that certain yeasts and bacteria can reuse organic acids as a carbon source when available sugars are depleted, resulting in a temporary reduction in measurable acidity [29]. However, in later stages, yeast’s autolysis releases nutrients that can regrow AAB, resuming the acid production if residual ethanol or sugar remains in the matrix. Additionally, SCOBY has a high-water absorption capacity, which could conceal a portion of the organic acids, thus reducing the acidity. As fermentation progresses, these acids may gradually re-enter the liquid, contributing to the observed increase in titratable acidity, as it was evidenced in the present study. As shown in Figure 1, the total acidity was associated with the treatment at 28 °C for 15 days, suggesting that longer fermentation times and higher temperatures enhance the acid production in kombucha.

### 3.8. Color Analysis

The color parameters shown in Table 2 exhibited measurable variations, some of which were statistically significant (*p* < 0.05). For L* (lightness), the samples at 23 °C on days 7, 11, and 15 were not significantly different. In contrast, at 25 °C, the L* value on day 15 was significantly lower than on day 7 and 11, (18.20 vs. 19.73 and 19.91, respectively), confirming a perceptible darkening of the beverage during the fermentation process. At 28 °C, no significant changes in lightness during the different fermentation times were observed. As shown in Figure 1, the vector lies near the center, slightly associated with mid-duration fermentation.

For a* (red–green axis) on Table 2, a significant increase in redness was observed at 23 °C on day 11, differing from days 7 and 15 (9.04 vs. 7.29 and 7.78, respectively). At 25 °C, no significant changes were observed in a* for all fermentation times. At 28 °C, there was greater variability on day 11, with a value of 8.67, which was statistically different from days 7 and 15, with values of 7.76 and 7.59, respectively. As shown in Figure 1, the a* vector leaned toward mid-duration fermentation treatment, similar to the behavior found for the L* vector in the PCA.

For b* (yellow–blue axis) on Table 2, no significant changes were observed for all fermentation times at 23 °C. However, at 25 °C, a significant decrease was shown on day 11 with a value of 5.26 from a value of 7.08 on day 7. The b* values then showed a moderate increase by day 15, with a value of 6.15. At 28 °C, no significant changes were observed for all fermentation times. As shown in Figure 1, the b* variable was positioned near 25 °C for 15 days of treatment, indicating an increase in yellowness for those kombucha products.

Although CIELAB parameters capture measurable color shifts, this study did not quantify polyphenols or their transformation products. Therefore, interpretation of color changes is limited to colorimetric trends, and future work integrating chromatographic phenolic profiling would strengthen the link between fermentation conditions and polyphenol development for color.

In the experiment conducted by Phung et al. (2023), kombucha produced with black tea showed higher L*, a*, and b* values, as pigmentation in kombucha may vary with the ratios of raw materials used [30]. In contrast to the study, our results showed much lower values for the color parameters, indicating a darker appearance. This suggests that although both studies use black tea, different conditions may lead to pigment degradation, affecting color chroma and intensity.

Accordingly, the physicochemical responses measured in this work (pH, titratable acidity, organic acid levels, viscosity, °Brix, and color) are interpreted as a downstream outcome of SCOBY metabolism. Nevertheless, because microbial populations were not tracked during fermentation, links between specific microbial groups and measured responses are interpretive rather than conclusive evidence of microbial dynamics. Further microbial analysis is needed to confirm these findings.

### 3.9. pH RSM Model

Table 3 shows the second-order model equation for the pH of the kombucha samples (3.05 − 0.17X_1_ − 0.06X_2_ − 0.00X_1_X_2_ + 0.13X_1_X_1_ + 0.03X_2_X_2_), where X_1_ is the fermentation time (days) and X_2_ is the fermentation temperature (°C). A highly significant, linear decrease in pH was observed during fermentation (X_1_ = −0.17, X_2_ = −0.06, *p* < 0.01), indicating that each additional day and each increase in temperature significantly decreased the pH values in all kombucha products. As shown in Table 3, the interaction effects (X_1_X_2_ = −0.00, *p* = 1.00) indicate that the model followed a strictly non-significant interaction, suggesting that time and temperature had effects on pH that were independent and additive.

**Figure 2:**
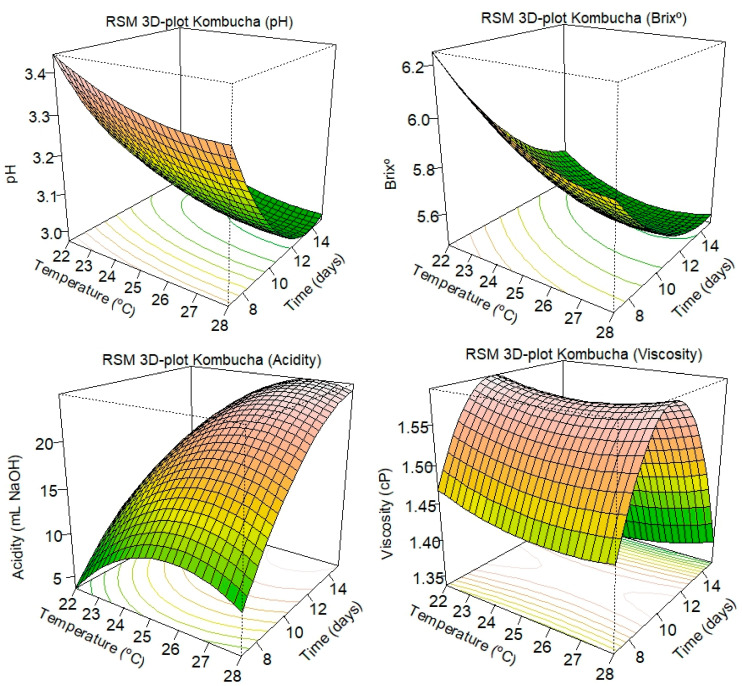
Response surface methodology (RSM) 3D plots for pH, Brix, acidity, and viscosity of the kombucha products.

The quadratic term on the pH RSM equation (X_1_X_1_ = +0.13, X_2_X_2_ = +0.03, *p* < 0.01) reveals a slowing of the pH decrease at longer times and higher temperatures of fermentation, indicating a self-inhibitory effect on these extended parameters. In other words, the pH behavior is not purely linear, but it does have a significant curvature component. A positive quadratic time and temperature term indicates a U-shaped trend, where pH initially decreased but slightly increased in later fermentation times and higher temperatures, as shown in Figure 2.

The full second-order model with linear, interaction, and quadratic terms (*p* < 0.01), demonstrates that time and temperature are valid predictors of pH behavior. A significant lack-of-fit (*p* < 0.01), even when R^2^ is relatively high (R^2^ = 0.80, adj. R^2^ = 0.78), reveals that the model fails to capture certain systematic patterns. This is often due to the omission of other non-linear effects or higher-order interactions. In the study by Chong et al. (2024), third-order polynomial models were applied to relate pH to SCOBY biomass formation and acetic acid production [31]. These models achieved R^2^ values of 0.98 and 0.97, respectively. This demonstrates that incorporating cubic effects enabled improved predictions of fermentation outcomes. However, including more terms in the equation can cause overfitting and multicollinearity.

### 3.10. °Brix RSM Model

Table 3 shows the second-order model equation for the °Brix of the kombucha samples (5.61 − 0.25X_1_ − 0.13X_2_ + 0.06X_1_X_2_ + 0.12X_1_X_1_ + 0.09X_2_X_2_); where X_1_ is the fermentation time (days), and X_2_ is the fermentation temperature (°C). The linear coefficients (X_1_ = −0.25, X_2_ = −0.06, *p* < 0.01) were significant in the model, indicating that both parameters (time and temperature) contributed to the decrease in soluble solids. However, the positive quadratic terms (X_1_X_1_ = +0.12, X_2_X_2_ = +0.09, *p* = 0.11) and a positive interaction term (X_1_X_2_ = +0.06, *p* = 0.20) were not statistically significant. Although the model is statistically significant (*p* < 0.01), the lack-of-fit test (*p* < 0.01) suggests that the current quadratic form does not fully encompass the total variability in the responses, indicating that the model fails to fully capture the observed patterns (Figure 2). This, together with a relatively low adjusted coefficient of determination (R^2^ = 0.54 and R^2^ adj. = 0.49), suggests that other relevant variables might have contributed to changes in total soluble solids in the kombucha samples.

### 3.11. Acidity RSM Model

Table 3 shows the second-order model equation for the acidity of the kombucha samples (20.16 + 6.51X_1_ + 3.72X_2_ + 1.48X_1_X_2_ − 3.39X_1_X_1_ − 4.42X_2_X_2_); where X_1_ is the fermentation time (days) and X_2_ is the fermentation temperature (°C). The linear effects on acidity of the fermentation time and temperature (X_1_ = +6.51, X_2_ = +0.03, respectively, *p* < 0.01) were significant in the RSM model. The positive coefficients confirm that acid content increased with increased time and temperature. The interaction term (X_1_X_2_ = +1.48, *p* = 0.10) was not significant, suggesting that time and temperature acted independently on the total acidity of the kombucha samples.

The quadratic terms (X_1_X_1_ = −3.39, X_2_X_2_ = −4.42, *p* < 0.01) were significant, and their negative coefficients indicate that curvature behaviors were shown as the increase in acidity slowed down as fermentation times were extended and temperatures increased. This can be observed in Figure 2, where the RSM 3D plot of acidity exhibits a plateau as both variables approach peaks in their responses. The lack-of-fit test was not statistically significant (*p* = 0.19), suggesting that the model was adequate to fit the variability in the acidity data. Quadratic models produce smooth surfaces; however, they may fail to detect abrupt changes, such as those shown in kombucha samples produced at 28 °C for 11 days of fermentation (Table 1). Nevertheless, the RSM model exhibited a strong predictive power, with an R^2^ of 0.73 and an adjusted R^2^ of 0.70.

### 3.12. Viscosity RSM Model

Table 3 shows the second-order model equation for the viscosity of the kombucha samples (1.57 − 0.05X_1_ − 0.00X_2_ + 0.01X_1_X_2_ − 0.20X_1_X_1_ + 0.2X_2_X_2_); where X_1_ is the fermentation time (days), and X_2_ is the fermentation temperature (°C). Both time and temperature linear effects (X_1_ = −0.05, X_2_ = −0.00, respectively, *p* < 0.01) were statistically significant on viscosity. However, the temperature coefficient was negligible in size compared to its time counterpart. The interaction between time and temperature (X_1_X_2_ = +0.01, *p* = 0.38) was not statistically significant, suggesting that time and temperature affected viscosity independently.

On the other hand, the quadratic terms in the model were significant (X_1_X_1_ = −0.20, X_2_X_2_ = +0.02; *p* < 0.01), showing that viscosity increased at shorter fermentation times but then decreased after prolonged fermentation. This behavior is illustrated in Figure 2, where viscosity values peaked at a medium fermentation time (around 11 days). Although the total model is statistically significant (*p* < 0.01), the lack-of-fit test (*p* < 0.01) indicates that the quadratic model does not fully capture the behavior in the viscosity values. The model showed an R^2^ of 0.69 and an adjusted R^2^ of 0.65, indicating a moderate fit for most of the variability. However, it suggests that the model could be improved by including a cubic effect term or by incorporating additional variables into the experiment.

## 4. Conclusions and Limitations

This study showed that fermentation time and temperature significantly affected the physicochemical characteristics of kombucha, with higher temperatures and longer durations associated with greater acidification, increased acetic acid content, and increased SCOBY biomass development, while lower temperatures favored lactic acid accumulation and higher °Brix values. Nonlinear changes in viscosity and titratable acidity may be related to differences in pellicle development and overall fermentation conditions across treatments; however, because BC and microbial composition were not quantified, these patterns cannot be attributed to temperature-dependent cellulose synthesis or specific microbial interactions within the fermentation matrix. Response surface modeling (RSM) indicated that time and temperature exerted linear and quadratic effects on pH, acidity, °Brix, and viscosity, highlighting a complex but quantifiable behavior of the fermentation system. Overall, these findings support the use of time–temperature controls as a practical basis for optimizing fermentation parameters to achieve consistent, desirable quality attributes in commercial kombucha production, while also highlighting key measurements needed in future studies to support specific strain activity.

Regarding limitations, this study focused on physicochemical outcomes as a function of fermentation time and temperature during active SCOBY fermentation. Microbial composition was available only as supplier-provided baseline information, and temporal microbial tracking was not performed; therefore, interpretation regarding microbial activity is inferential. In addition, uninoculated or SCOBY-inactivated controls were not included, which limits the ability to fully separate biological from abiotic contributions to certain responses. Finally, BC content and tea polyphenolic profiles were not measured, making the SCOBY weight and color change results difficult to interpret. Future studies integrating microbial tracking, abiotic controls, cellulose quantification, and polyphenol profiling would strengthen causal interpretation.

## Figures and Tables

**Table 1:** Physico-chemical parameters of the kombucha products.

Temp. (°C)	Time (Days)	pH	Viscosity (cP)	Acetic Acid (%)	Lactic Acid (%)	Brix (°)	Diff. (g)	Acidity (mL)
23	7	3.47 ± 0.01 ^a^	1.45 ± 0.05 ^cd^	12.70 ± 0.33 ^ab^	4.23 ± 0.06 ^bc^	6.15 ± 0.05 ^ab^	−7.62 ± 4.01 ^e^	9.30 ± 0.77 ^d^
	11	3.07 ± 0.02 ^def^	1.40 ± 0.00 ^cde^	11.97 ± 0.20 ^c^	5.46 ± 0.91 ^ab^	5.85 ± 0.05 ^abc^	8.98 ± 4.81 ^bc^	4.85 ± 0.27 ^d^
	15	3.15 ± 0.03 ^cd^	1.45 ± 0.05 ^cd^	11.50 ± 0.33 ^c^	5.79 ± 0.09 ^a^	6.20 ± 0.00 ^a^	15.92 ± 6.61 ^ab^	20.75 ± 5.20 ^ab^
25	7	3.36 ± 0.02 ^ab^	1.65 ± 0.05 ^a^	13.23 ± 0.27 ^a^	1.79 ± 0.22 ^e^	5.45 ± 0.05 ^cd^	−3.68 ± 2.38 ^de^	12.75 ± 2.14 ^bcd^
	11	3.06 ± 0.06 ^def^	1.45 ± 0.05 ^cd^	11.67 ± 0.10 ^c^	2.73 ± 0.27 ^de^	5.75 ± 0.16 ^bcd^	−4.18 ± 4.04 ^de^	11.70 ± 1.86 ^cd^
	15	3.00 ± 0.08 ^ef^	1.60 ± 0.00 ^ab^	12.17 ± 0.08 ^bc^	4.05 ± 0.12 ^bcd^	5.40 ± 0.33 ^d^	3.96 ± 2.21 ^cd^	19.20 ± 5.04 ^abc^
28	7	3.28 ± 0.04 ^bc^	1.30 ± 0.00 ^e^	11.60 ± 0.00 ^c^	3.89 ± 1.85 ^cd^	5.50 ± 0.00 ^cd^	−3.92 ± 3.00 ^de^	23.10 ± 4.27 ^a^
	11	3.10 ± 0.18 ^de^	1.50 ± 0.11 ^bc^	12.00 ± 0.00 ^c^	4.41 ± 0.68 ^abc^	5.65 ± 0.05 ^cd^	10.50 ± 8.00 ^bc^	7.80 ± 0.33 ^d^
	15	2.96 ± 0.00 ^f^	1.35 ± 0.05 ^de^	12.00 ± 0.88 ^c^	2.30 ± 0.14 ^e^	5.75 ± 0.49 ^bcd^	23.68 ± 9.82 ^a^	25.15 ± 9.15 ^a^

Temp. = Temperature; °C = Degrees Celsius. ^abcdef^ Means with different letters are statistically different (*p* < 0.05). Acidity (mL) was obtained by the method of NaOH titration. Diff. = Difference (g) is the weight difference between the SCOBY before and after fermentation of the kombucha.

**Table 2:** Color analysis of kombucha products.

Temperature (°C)	Time (Days)	L*		a*		b*	
23	7	18.69 ± 0.39	^bc^	7.29 ± 0.50	^c^	5.95 ± 0.21	^abc^
	11	19.32 ± 0.66	^abc^	9.04 ± 0.08	^a^	5.95 ± 1.18	^abc^
	15	19.71 ± 0.14	^ab^	7.78 ± 0.74	^bc^	5.68 ± 0.15	^bc^
25	7	19.73 ± 0.01	^ab^	8.00 ± 0.94	^bc^	7.08 ± 0.19	^a^
	11	19.91 ± 0.82	^a^	7.51 ± 0.08	^c^	5.26 ± 0.28	^c^
	15	18.20 ± 0.38	^c^	7.96 ± 0.25	^bc^	6.15 ± 0.30	^abc^
28	7	19.21 ± 0.19	^abc^	7.76 ± 0.05	^bc^	6.07 ± 0.11	^abc^
	11	18.84 ± 1.10	^abc^	8.67 ± 0.02	^ab^	5.75 ± 1.05	^bc^
	15	19.02 ± 0.80	^abc^	7.59 ± 0.44	^c^	6.55 ± 0.99	^ab^

°C = Degrees Celsius. ^abc^ Means with different letters are statistically different (*p* < 0.05).

**Table 3:** Regression effects and parameter estimate of predictive regression models for pH, Brix, acidity, and viscosity of the kombucha products using response surface methodology (RSM).

Parameter	Regression Effects (*p* > F) *				Residual (*p* > F)	Model Fitness	
	First Order	Interaction	Quadratic	Total Model	Lack of Fit	R-Square	Adj. R-Square
pH	***<0.01***	1.00	***<0.01***	***<0.01***	***<0.01***	0.80	0.78
Brix (°)	***<0.01***	0.20	0.11	***<0.01***	***<0.01***	0.54	0.49
Acidity (mL)	***<0.01***	0.10	***<0.01***	***<0.01***	0.19	0.73	0.70
Viscosity (cP)	***<0.01***	0.38	***<0.01***	***<0.01***	***<0.01***	0.69	0.65
**Parameter**	Parameter Estimates **						
pH	3.05 − 0.17*X*_1_ − 0.06*X*_2_ − 0.00*X*_1_*X*_2_ + 0.13*X*_1_*X*_1_ + 0.03*X*_2_*X*_2_						
Brix (°)	5.61−0.25*X*_1_−0.13*X*_2_ + 0.06*X*_1_*X*_2_ + 0.12*X*_1_*X*_1_ + 0.09*X*_2_*X*_2_						
Acidity (mL)	20.16 + 6.51*X*_1_ + 3.72*X*_2_ + 1.48*X*_1_*X*_2_ − 3.39*X*_1_*X*_1_ − 4.42*X*_2_*X*_2_						
Viscosity (cP)	1.57 − 0.05*X*_1_ − 0.00*X*_2_ + 0.01*X*_1_*X*_2_ − 0.20*X*_1_*X*_1_ + 0.02*X*_2_*X*_2_						

* Effects were considered significant when *p* > F was < 0.05 (***bolded and italicized probabilities***). First order represents the two main effects in the model (time and temperature, degrees of freedom = 2), interaction represents the two-way interaction of the main effects (degrees of freedom = 1), and quadratic represents the quadratic effect of the main effects (degrees of freedom = 2), and total model represents all the regression effects altogether (degrees of freedom = 5). ** *X*_1_ = Time of fermentation (days), and *X*_2_ = Temperature of fermentation (°C).

## Data Availability

The original contributions presented in this study are included in the article. Further inquiries can be directed to the corresponding author.
